# Supplementary material for: Immobilization of Alendronate on Zirconium Phosphate Nanoplatelets
Source: Nanomaterials (Basel). 2023 Feb 15;13(4):742. doi: 10.3390/nano13040742 (PMC9965588; doi:10.3390/nano13040742)
Supplement: Supplementary file 1 [file nanomaterials-13-00742-s001.zip › nanomaterials-2201041-supplementary.pdf]

nanomaterials

Supplementary Materials

Immobilization of Alendronate on Zirconium Phosphate Nanoplatelets

Anna Donnadio [1,2,*], Geo Paul [3], Marianna Barbalinardo [4], Valeria Ambrogi [1], Gabriele Pettinacci [1], Tamara Posati [5], Chiara Bisio [3,6], Riccardo Vivani [1,2] and Morena Nocchetti [1,2,*]

[1] Department of Pharmaceutical Sciences, University of Perugia, via del Liceo 1, 06123 Perugia, Italy
[2] CEMIN-Centro di Eccellenza Materiali Innovativi Nanostrutturati, University of Perugia, via Elce di Sotto 8, 06123, Perugia, Italy
[3] Department of Sciences and Technological Innovation, University of Piemonte Orientale, "A. Avogadro", viale T. Michel 11, 15121 Alessandria, Italy
[4] CNR-ISMN, Via P. Gobetti 101, 40129 Bologna, Italy
[5] CNR-ISOF, Via P. Gobetti 101, 40129 Bologna, Italy
[6] CNR-Istituto di Scienze e Tecnologie Chimiche "Giulio Natta", Via C. Golgi 19, 20133, Milano, Italy
* Correspondence: anna.donnadio@unipg.it (A.D.); morena.nocchetti@unipg.it (M.N.)

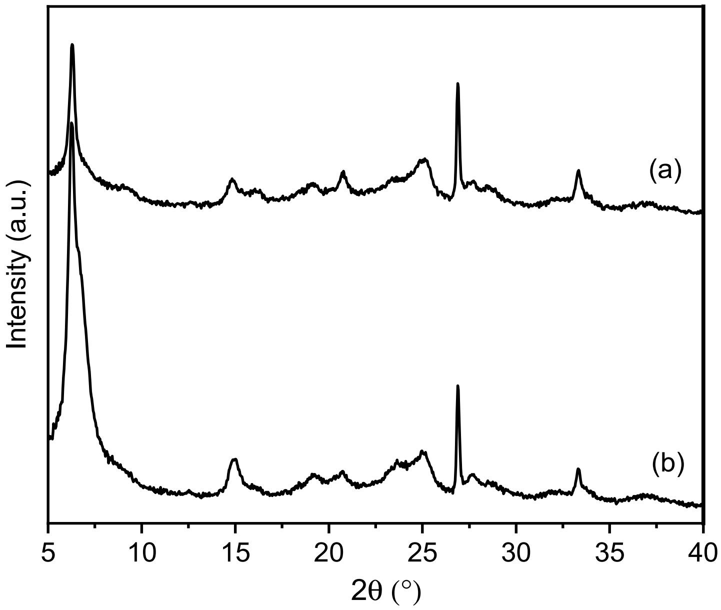

Figure S1. XRPD patterns of γZP05 (a) and γZP1 (b) washed with HCl and conditioned over P_2O_5.

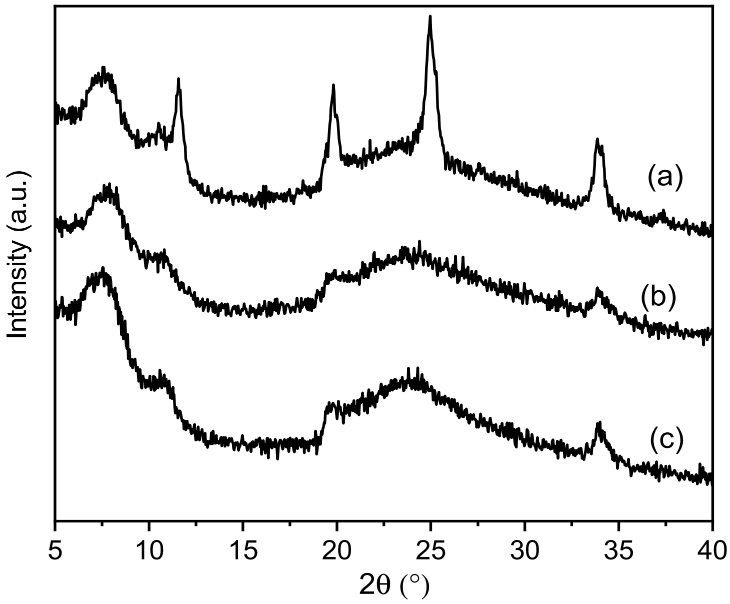

Figure S2. XRPD patterns of samples washed with HCl and conditioned over P_2O_5: αZP05 (a), αZP1 (b) and αZP2 (c).

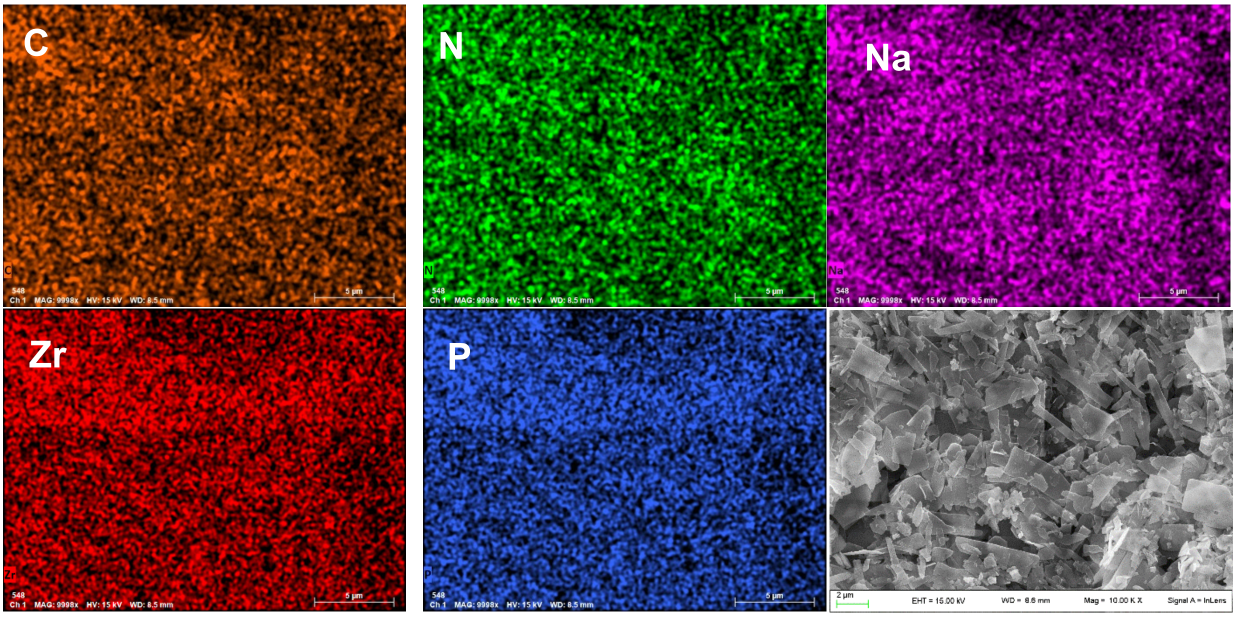

Figure S3. EDX analysis of γZP1.

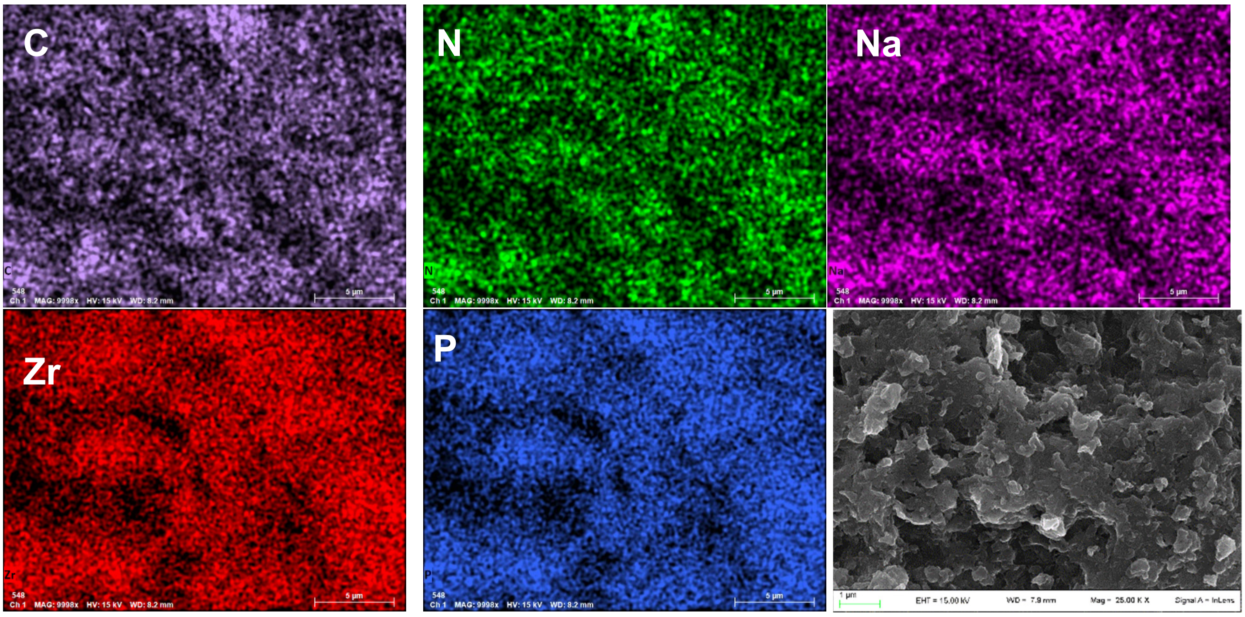

Figure S4. EDX analysis of αZP1.
